# Factors influencing pedestrian bridge use: a self-report study

**Published:** 2019-08

**Authors:** Mohammad Saadati, Elnaz Hemmatie, Monireheh Edalatzadeh, Tina Hosseinpor, Tahereh Naglisar, Hassan Moradie

**Affiliations:** ^*a*^Road Traffic Injury Research Center, Tabriz University of Medical Sciences, Tabriz, Iran.; ^*b*^Tabriz Health Center, Tabriz University of Medical Sciences, Tabriz, Iran.; ^*c*^Sports and Youth Office, Tabriz, Iran.

## Abstract

**Background:**

Pedestrian bridges are a safe tool for movement of pedestrian. The use of these bridges are affected by some factors related to the individuals, environment, bridge and etc. the aim of this study was to identify the factors influencing use/nonuse of pedestrian bridge in Tabriz, Iran.

**Methods:**

Using cross-sectional approach, we have conducted a study in Tabriz, 2019. Using a pilot study (n=30) data and Cochrane formula, the sample size was estimated to be 360. Sampling was done using simple random sampling method. Two types of the bridges with/whiteout electrical stair were included in the study. Sample allocation was done based on the crowdedness of the bridges. A valid questionnaire (CVI= 0.78, α=0.75) was used for data collection. Data was analyzed using SPSS 21.

**Results:**

Totally 358 people were participated with an average age of 29 years. About 36% were female and 37% have BSc level of education. Moe than 72% of the participants have driving license and about a quarter of them had a crash history. Nearly 17% declared that they use pedestrian bridge sometimes or less. 43% believed that bridge using is only in crowded streets is necessary. Low safety of the bridges, bad appearance and low lightness of the bridges were among the barriers. Also participants declared that when they are with a child, they are more encouraged to use the bridge.

**Conclusions:**

Pedestrians attitude about the bridges safety, the bridges appearance and lighting were identified as the factors influencing bridges use. Designing pedestrian bridges using artistic principles will create more sense of safety which facilitates the bridges using.

**Keywords:**

Traffic safety, Pedestrian, Bridge

